# Molecular characteristics of the full-length genome of occult hepatitis B virus from blood donors in China

**DOI:** 10.1038/s41598-022-12288-0

**Published:** 2022-05-17

**Authors:** Min Wang, Ru Xu, Jieting Huang, Qiao Liao, Xi Tang, Zhengang Shan, Huishan Zhong, Xia Rong, Yongshui Fu

**Affiliations:** 1 Institute of Clinical Blood Transfusion, Guangzhou Blood Center, Guangzhou, Guangdong China; 2 The Key Medical Laboratory of Guangzhou, Guangzhou, Guangdong China; 3 Department of Infectious Diseases, Foshan First People′s Hospital, Guangzhou, Guangdong China; 4 School of Laboratory Medicine and Biotechnology, Southern Medical University, Guangzhou, Guangdong China

**Keywords:** Genetics, Microbiology

## Abstract

The characteristics of a large sample size of the full-length genome of occult hepatitis B virus (HBV) infection (OBI) have not been extensively explored in China. Voluntary blood donors who were HBsAg-negative/HBV NAT-positive (HBsAg−/HBV NAT+) were identified by blood screening and recruited. Blood samples were tested for HBV serologic markers, viral loads, and PCR to identify OBI. HBV full-length genomes were obtained by amplifying two fragments using nested PCR. The characterization of OBI strains was based on sequence analyses compared with HBsAg+ strains obtained from the same donor population. Of the 50 full-length genomes of 172 identified OBI strains, 33 were classified as genotype B (OBI_B_) and 17 strains as genotype C (OBI_C_). Significantly higher nucleotide variabilities were observed in the Pre-S2/S promoter region (SP2) and core upstream regulatory sequence (CURS) in OBI_B_ than in their HBsAg+ controls (*P* < 0.05). Both OBI_B_ and OBI_C_ showed higher amino acid (aa) variabilities in Pol and Pre-S/S regions than their controls (*P* < 0.05). In addition, 19 novel OBI-related mutations were found spanning the four open reading frames (ORFs) of the HBV genome. Four novel deletions and one novel insertion were also found in OBI_C_ strains. Several novel OBI-related mutations spanning the four ORFs of the virus were identified by characterizing a large sample size of the full-length OBI genome, which may affect the production of HBsAg and contribute to the occult infection of HBV.

## Introduction

Implementation of serological testing, especially in voluntary blood donors, is critical for controlling the transmission of the hepatitis B virus (HBV)^1^. After the introduction of the nucleic acid test (NAT) in blood screening, the risk of HBV transmission by transfusion has been significantly decreased because blood components with HBsAg-negative /HBV NAT-positive (HBsAg−/HBV NAT+), which were missed by serological testing, can be identified by NAT^2–5^. It has been reported that the majority of HBsAg−/HBV NAT+ cases were occult HBV infection (OBI)^6,7^. The definition of OBI is the presence of replication-competent HBV genome in the blood and/or liver of individuals who test negative for HBsAg by the currently available testing methods^8^. Defining the epidemiology of OBI can be difficult because it relies on the sensitivity of HBsAg and HBV DNA assays. OBI is the potential risk of HBV transmission through blood transfusion, and organ transplantation, as well as from occult infected mothers to newborns^9–11^. In low- and middle-income countries that anti-HBc and/or NAT tests have not been implemented, HBV transmission from OBI blood donors remains a major health issue^8^. The biochemical and clinical symptoms of most OBIs are not serious, but serious liver diseases can also occur^12^.

The virological features and the mechanisms of OBI remain unclear. OBI has been related to the HBV S protein mutations in vitro^13–16^. Other reasons leading to OBI include genomic regulatory regions mutations that may negatively affect viral replication^17^ and incomplete control of HBV under the host immune system^18–20^. Although there have been many studies on Pre-S/S mutation in OBI^14,20,21^, few studies reported full genomes of OBI, or the sample size was limited^22^. Here, we aimed to characterize OBI from blood donors in Guangdong, China. The sample size in this study was large, because we got 50 full-length genome sequences of OBI. The specific aim of this study was to report the more comprehensive and detailed serological and molecular characteristics of OBI in China to assess whether specific viral mutations could characterize OBI.

## Results

### Sample classification and characterization

From March 2015 to May 2017, a total of 554,154 blood donors were screened and 1793 blood donors were HBsAg negative and multiplex NAT positive. Among them, 1407 were further determined using HBV Discriminatory Assay, and 428 were found to be HBV NAT positive. Among 428 HBsAg−/HBV NAT+ samples, 212 were randomly selected as the study population. Among them, 77 (36.32%) were reactive for anti-HBc and anti-HBs, 105 (49.53%) carried anti-HBc only, 13 (6.13%) were reactive for anti-HBs only, and 17 (8.02%) were non-reactive for anti-HBc and anti-HBs (Table 1).

**Table 1:** Molecular and serological confirmation of hepatitis B virus (HBV) infection in NAT-positive blood donor samples.

Serological parameters	Confirmed as HBV DNA positive^δ^, *n* (%)			Not confirmed as HBV DNA positive^ε^, *n* (%)	
	OBI	Other^φ^	Not classifiable		Total
Anti-HBc + /anti-HBs +	70 (90.91%)	0	0	7 (9.09)	77 (36.32)
Anti-HBc + /anti-HBs-	102 (97.14)	0	0	3 (2.86)	105 (49.53)
Anti-HBc-/anti-HBs +	0	2 (15.39)	10 (76.92)	1 (7.69)	13 (6.13)
Anti-HBc-/anti-HBs-	0	4 (23.53)	12 (70.59)	1 (5.88)	17 (8.02)
Total	172 (81.13)	6 (2.83)	22 (10.38)	12 (5.66)	212

^δ^Samples positive with Q-PCR or nested PCR tests were considered HBV DNA positive.

^ε^Samples negative with Q-PCR and nested PCR assays were considered HBV DNA not confirmed.

^φ^These 6 samples were positive for HBV DNA (qPCR or nested PCR positive) and negative for anti-HBc, however, these follow-up samples were negative for HBV DNA by NAT, qPCR and nested PCR assays. Index sample 73, 368, 987 contained 29.3 IU/L, 84.3 IU/L, < 2 IU/L of anti-HBs but the first follow-up sample contained 239.1 IU/L, 75.7 IU/L, 69.5 IU/L of anti-HBs, respectively. The rest three samples were anti-HBs negative but the follow-up samples contained > 1000 IU/L of anti-HBs. These index samples with non-reactive for anti-HBc were classified as recent HBV infection, and acute resolving may happen to these 6 blood donors.

**Figure 1:**
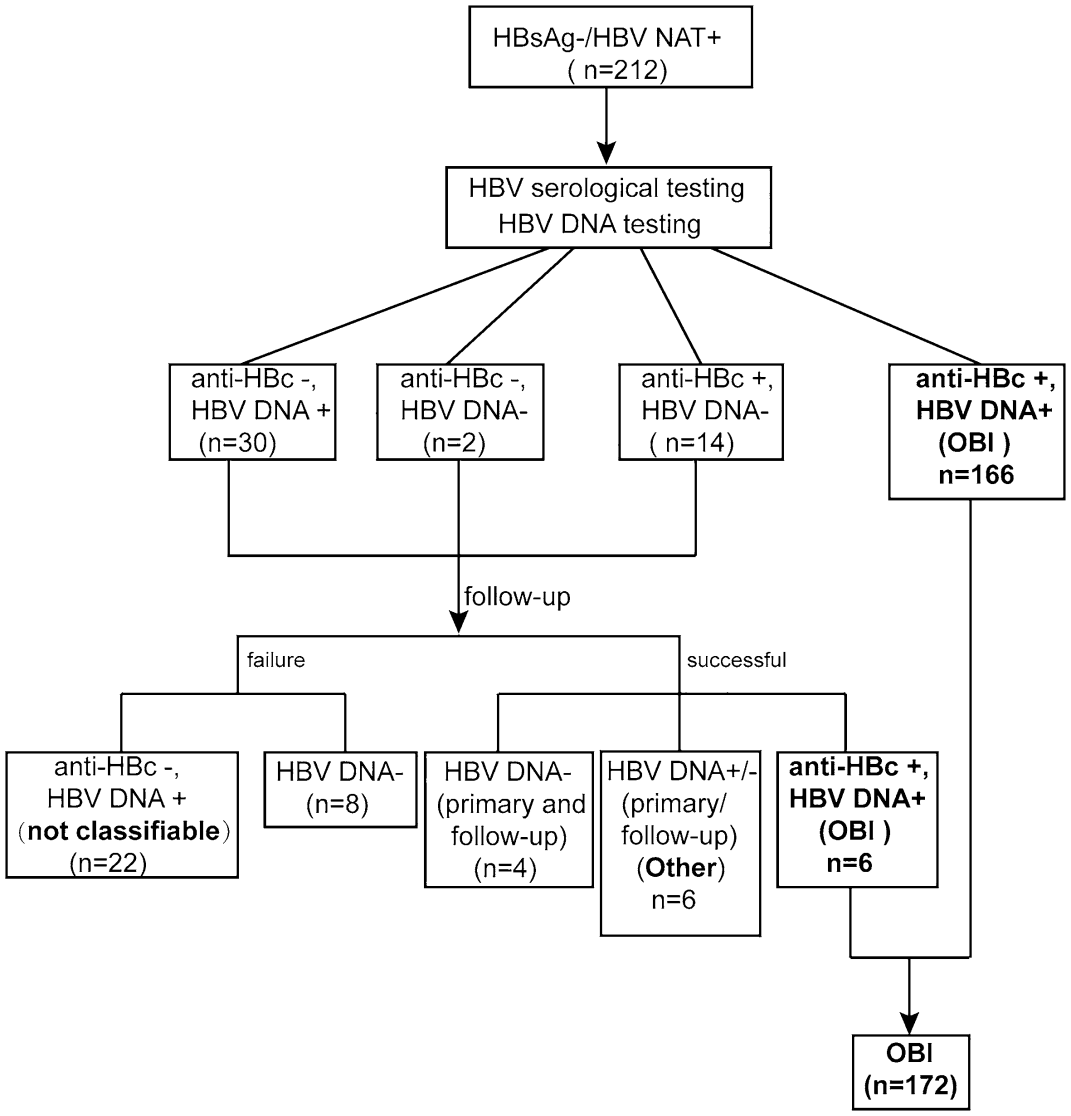
Detection and classification of HBV infection in blood donors (including follow-up samples) with HBsAg−/HBV NAT+. HBV serological testing was performed by electrochemiluminescence immunoassay (ECLIA) for HBsAg, anti-HBs, anti-HBc, HBeAg, and anti-HBe. In addition, HBV DNA testing was performed by real-time quantitative polymerase chain reaction (Q-PCR) and PCR for BCP/PC gene and long fragment. Six samples were positive for HBV DNA (Q-PCR or nested PCR positive) and negative for anti-HBc; however, in follow-up analyses, these samples were negative for HBV DNA by NAT, Q-PCR, and nested PCR assays. “failure” meant that we did not get the followed-up samples, and “successful” meant that we got the followed-up samples successfully.

In order to determine OBI, the existence of HBV DNA in the 212 HBsAg−/HBV NAT+ subjects on both primary donation and follow up samples were confirmed by Q-PCR and nested PCR. A total of 200 samples were considered HBV DNA positive, including 172 OBI, 6 "other" for various specific features (which can be recent HBV infection and acute resolving may happen), 22 unclassified samples that were anti-HBc negative could not be differentiated between seronegative primary or transient OBI and window period because of lack of follow-up, and 12 samples negative by Q-PCR and nested PCR assays were considered HBV DNA not confirmed (Table 1 and Fig. 1). The distribution of viral loads of the 200 HBV DNA positive subjects was shown in supplementary Table S1.

Among the 172 OBI donors, there were 137 males and 35 females. The median age was 44 years old. The values of ALT less than or equal to 50 U/L are defined as normal no matter male or female according to the manufacturer's instructions (Alanine aminotransferase Reagent Kit, Shanghai Huashi Asia–Pacific Biopharmaceutical Co., Ltd.). All of them had normal ALT levels. Their viral loads ranged between unquantifiable and 4,667 IU/ml (median, 71.15 IU/ml). The OBI samples were further classified by serological markers: 70 (40.70%) samples were reactive for anti-HBc and anti-HBs, and 102 (59.30%) carried anti-HBc only (Table 1).

**Figure 2:**
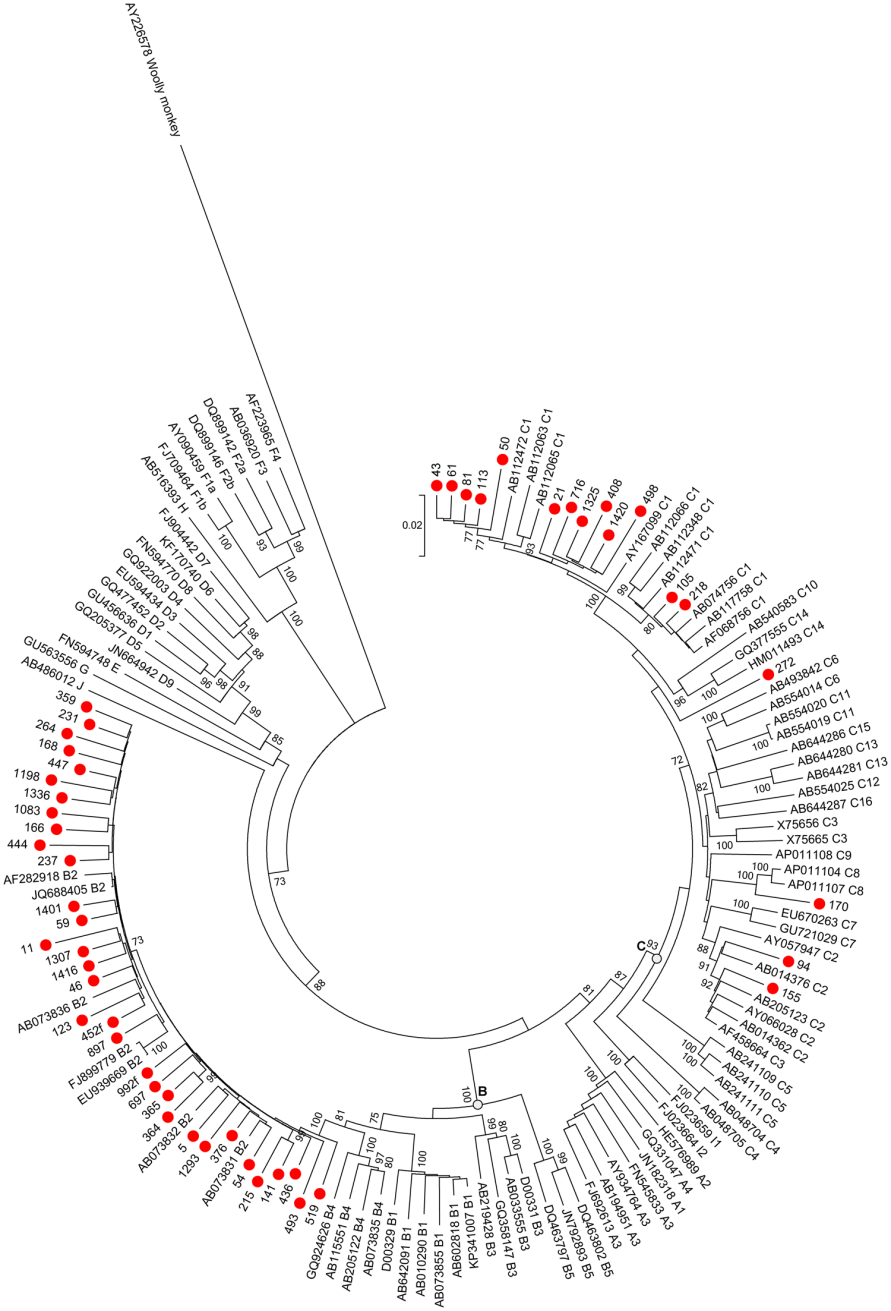
Estimated maximum-likelihood phylogeny for full-length genome sequences of OBI strains. Red circles indicate sequences from OBI strains in this study, and the rest indicate reference sequences of proposed genotypes and subgenotypes. Bootstrap analysis values (> 70%) are displayed on the branches. The bars at the middle top of the figure show the scale in nucleotide substitutions per site. AY226578: from Woolly monkey as an out-group.

Among 172 OBI samples, 129 BCP/PC and 50 long fragment sequences were amplified and sequenced; in total, 50 full-length genomes (including 3 HBV whole genomes minus 53 bp) were obtained, which classified 33 strains as genotype (gt) B (OBI_B_) and 17 strains as genotype C (OBI_C_) via phylogenetic analysis. All OBI_B_ strains were of subgenotype B2. Thirteen OBI_C_ strains were subgenotype C1, with 4 belonged to other subgenotypes of genotype C (Fig. 2). The mean viral load among OBI_B_ donors was 2.099 log10 IU/ml comparing to 1.915 log10 IU/ml among OBI_C_ donors (*P* = 0.743) using the non-parametric Mann–Whitney test (data not shown).

### Variability analysis of regulatory regions and core, X, envelope (Pre-S/S), and polymerase (Pol) proteins of occult HBV sequences

The nucleotide sequences of regulatory regions and amino acid sequences of ORFs from OBI_B_ and OBIc strains were compared to their respective control (HBsAg+) sequences. Regulatory regions of HBV, including enhancer (ENH), promoters for Pre-S1 (SP1), Pre-S2/S (SP2), Core (BCP), and X (XP) proteins, core upstream regulatory sequence (CURS), and direct repeat sequences (DR) were analyzed. DR1 and DR2 were conserved. Significantly higher variabilities (*P* < 0.05) were observed in SP2 and CURS regulatory regions in OBI_B_ strains than in their controls. In contrast, SP1 was less variable in OBI_B_ than controls (Table 2).

**Table 2:** Intergroup variability analyses between OBI_B_ and HBsAg + strains^δ^.

	Genome region	Genotype B			Genotype C		
		OBI strains (*n* = 33)	HBsAg + strains (*n* = 58)	*p* value	OBI strains (*n* = 17)	HBsAg + strains (*n* = 23)	*p* value
Regulatory region sequence variability^ε^	ENH1	2 (0–5)	3(0–8)	0.622	2(0–13)	2 (0–10)	0.808
	SP1	8 (3–16)	10 (1–19)	0.016	11 (0–36)	7 (2–57)	0.302
	SP2	5(1–9)	3 (0–10)	** < 0.001**	8 (1–31)	5 (1–31)	0.134
	ENH2	4(0–6)	2 (0–9)	0.074	4 (1–12)	4 (0–8)	0.626
	CURS	2 (1–3)	1 (1–4)	**0.013**	2 (0–7)	1 (0–4)	0.401
	BCP	2 (0–5)^a^	2 (0–5)	0.099	3 (0–5)	3 (0–7)	0.665
	XP	2 (0–7)	2 (0–6)	0.188	3 (1–6)	2 (0–11)	0.062
	DR1	0 (0)	0 (0)	1.000	0 (0)	0 (0)	1.000
	DR2	0 (0)	0 (0)	0.684	0 (0)	0 (0)	1.000
Protein sequence variability^φ^	Core protein	4(0–11)	8 (0–15)	< 0.001	1 (0–9)	5 (0–14)	0.232
	X protein	4 (0–8)*	3.5 (0–8)	0.454	4 (1–6)	2 (0–10)	0.242
	Pol protein	18 (8–32)	15 (3–30)	**0.009**	24 (5–57)	12 (3–66)	**0.042**
	Pol (RT)	7 (3–20)	4.5(0–11)	** < 0.001**	9 (2–22)	3 (1–17)	**0.005**
	Pre-S/S protein						
	Pre-S1	3 (1–7)	1 (0–5)	** < 0.001**	2.5 (0–11)^η^	1 (0–13)	0.288
	Pre-S2	3 (0–7)	0(0–7)	** < 0.001**	2 (0–8)	1 (0–12)	**0.013**
	S	11 (3–26)	4 (0–10)	** < 0.001**	11.5 (0–29)^η^	2 (0–10)	**0.004**
	MHR	4 (0–16)	1 (0–5)	** < 0.001**	5 (0–17) ^b^	0 (0–3)	** < 0.001**

Significant diversities are indicated in bold. The numbers shown mean the number of substitutions compared to the consensus sequence.

^δ^Average intragroup variability was calculated as the number of nucleotide (regulatory region sequences) or amino acid (protein sequences) substitution differences between OBI and control sequences by HBV different genotype. The range for frequency of nucleotide or amino acid substitutions is in parentheses. *n*, number of complete sequences from OBI or control strains used in analysis (except as noted). The difference of mean diversity between OBI and control sequences was calculated as the *P* value by the non-parametric Mann–Whitney U test.

^ε^ENH1 (nt 1071–1238), enhancer I, ENH2 (nt 1627–1774), enhancer II; SP1 (nt 2219 to 2780) and SP2 (nt 2809 to 3152), promoters for Pre-S1 and Pre-S2/S; XP (nt 1239 to 1376), promoter for X region; CURS (nt 1643 to 1741), core upstream regulatory sequence; BCP (nt 1742 to 1849), basal core promoter, DR1 (nt1824–4834) and DR2 (nt1590–1600), direct repeat sequences.

^φ^MHR, major hydrophilic region of S protein; Pol, polymerase; Pol (RT), reverse transcriptase.

*n = 30 (3 HBV whole genome sequences minus 53 bp were excluded), ^η^*n* = 16 (2 sequences with stop codon mutations were excluded).

The variability of amino acid sequences of ORFs was analyzed using the same method. Amino acid variabilities were significantly higher in Pol and Pre-S/S protein of both OBI_B_ and OBI_C_ than their corresponding controls (Table 2).

### Mutation analysis on Pre-S/S, Pol, Pre-core/core, and X region between OBI strains and controls

**Figure 3:**
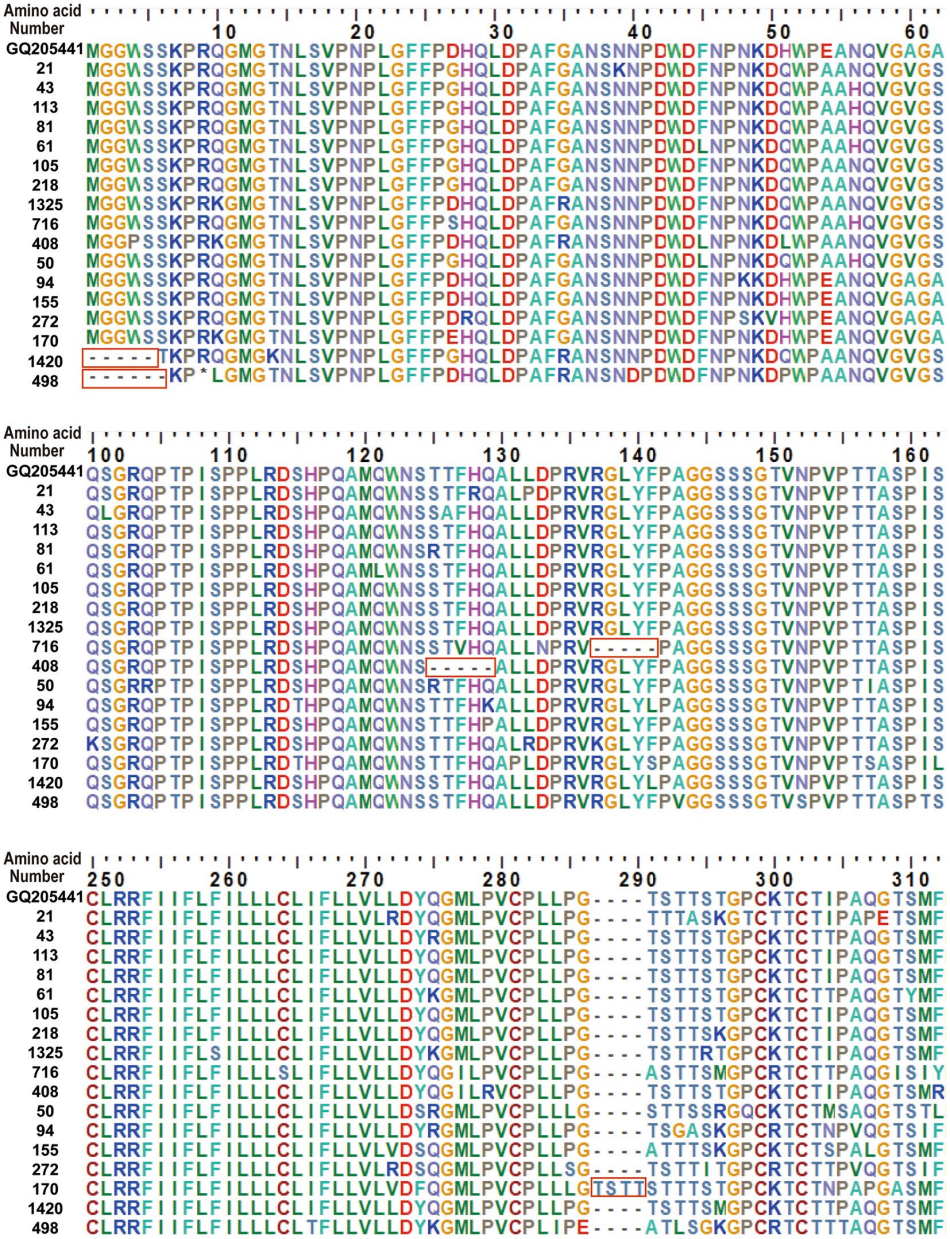
Amino acid location of deletions and insertion at the PreS/S region for gtC strains. Deleted or inserted amino acid sequences were labeled with long red boxes. GQ205441 was a reference sequence of genotype C.

Deletion and insertion were found in 4 cases and 1 case in OBI_C_ strain, respectively (Fig. 3). Samples 408 and 716 had amino acids 6–10 and 18–22 deleted in Pre-S2, respectively. Sample 498 and 1420 had amino acids 1–6 and 1–5 deleted in Pre-S1, respectively, which leads to start codon lost in Pre-S1. Sample 170 had 4 amino acids insertion (after aa112 in S gene), which was located in the major hydrophilic region (MHR). Five clones of each strain were sequenced and were all support the deletion/insertion (Supplementary Fig. S2). Deletions and insertion in this study have not been found in other studies^23–27^.

To identify OBI-related point mutations, the amino acid sequences of OBI_B_ and OBI_C_ strains (excluding strains with deletions and insertions) were compared with their corresponding controls. Forty-five OBI-related point mutations were identified in Pre-S1, Pre-S2, S, Core, and Pol genes, because their frequency in OBIs was significantly higher than that in the controls (*P* < 0.05; Table 3 and Supplementary Table S2). Among them, 26 mutations have been documented in previous studies (Supplementary Table S2)^14,16,20,28,29^, while the other 19 mutations were novel findings, including E39K/D and S101T in Pre-S1 gene, Q10R in Pre-S2 gene, P178Q, Q181R, and I226N in the S gene, T147A in Core gene in OBI_B_ and T118K in S gene in OBI_C_ (Table 3).

**Table 3:** Novel OBI-related mutations of HBV genome relating to the genotype B and C.

Region	Mutation	Frequency in OBI blood donors (%)	Frequency in HBs-positive blood donors (%)	*p*-Value (OBI vs HBsAg-positive)
**Gt-B**				
Pres1	E39K	8/33 (24.24)	3/58 (5.00)	0.004
	E39D	3/33(9.09)	0/58 (0)	0.023
	S101T	3/33 (9.09)	0/58 (0)	0.045
Pres2	Q10R	3/33 (9.09)	0/58 (0)	0.041
S	P178Q	3/33 (9.09)	0/58 (0)	0.038
	Q181R	4/33 (12.12)	0/58 (0)	0.014
	I226N	4/33 (12.12)	0/58 (0)	0.010
Core	T147A	10/33 (30.30)	6/58 (10.34)	0.023
Pol	G261R	4/33 (12.12)	0/58 (0)	0.040
	V281D	3/33 (9.09)	0/58 (0)	0.042
	R364K	4/33 (12.12)	0/58 (0)	0.015
	V458I	4/33 (12.12)	0/58 (0)	0.016
	N470H	3/33 (9.09)	0/58 (0)	0.045
	N572T	3/33 (9.09)	0/58 (0)	0.047
	Q613H	6/33 (18.18)	2/58 (3.45)	0.025
**Gt-C**				
S	**T118K**	5/15 (33.33)	0/23 (0)	0.003
Pol	R119L	9/12 (75.00)	7/23 (30.43)	0.030
	H472Q^δ^	5/12 (41.67)	0/23 (0)	0.002
	I615L	3/12 (25.00)	0/23 (0)	0.028

^δ^Mutation pH472Q is corresponding to sT118K in OBIc strains.

OBI-related mutations in Pol gene rarely reported before. Here we identified 10 novel mutations within Pol gene (Table 3). It should be noted that, since the reverse transcriptase (RT) of the Pol gene spanned the S region completely, any single nonsynonymous mutation may lead to changes in amino acid of both proteins, which consequently referred two mutations. For example, pR499Q (rtR153Q) and pH580Q (rtH234Q) corresponding to sG145R and sI226N/S in OBI_B_ strains, and pH468N (rtR122 Q) and pH472Q (rtH126Q) corresponding to sS114T and sT118K in OBI_C_ strains were found in this study (Table 3 and Supplementary Table S2).

**Figure 4:**
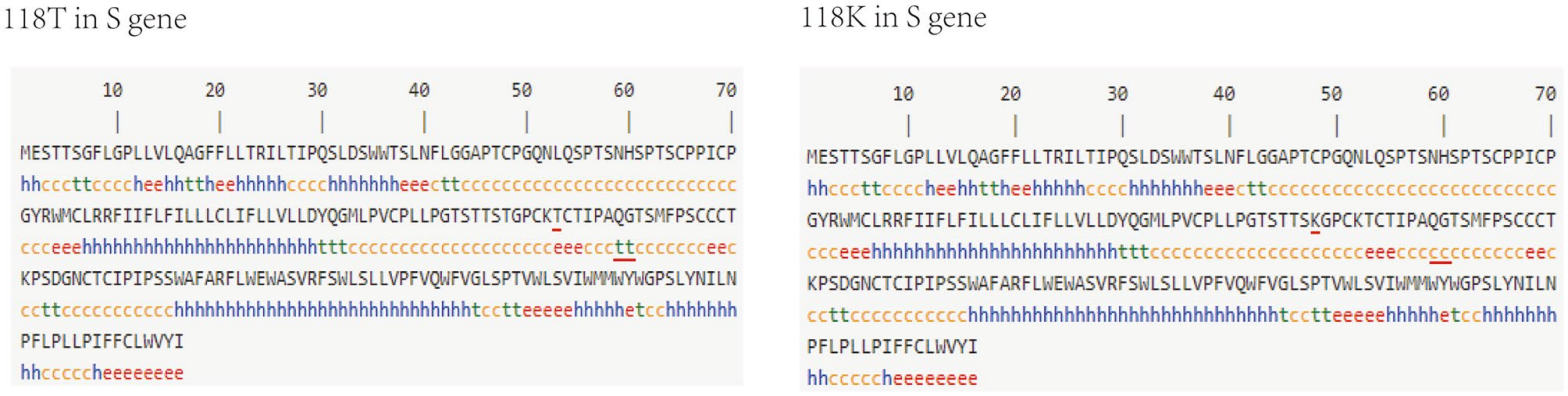
Prediction of the secondary structure of the S protein upon mutation sT118K in OBI_C_ strains. h (blue), alpha-helix; e (red), extended strand; t (green), beta-turn; c (yellow), random coil. Mutated amino acid and altered secondary structure were labeled with red glide line.

MHR is the most important antigenic determinant for HBV strains. Mutation sT118K in the MHR changed the secondary structure of the S protein (Fig. 4). This mutation had 15 instead of 17 amino acids in beta turns, and 97 instead of 95 amino acids in random coils. HBV DNA level in strains with OBI-related mutations and in strains without OBI-related mutations were compared, and HBV DNA level was significantly lower in strains with mutations sG145R (pR499Q) than in strains without the mutation, so was mutation sI226S in OBI_B_ strains (Supplementary Table S3).

Two mutations resulted in stop codon were found: aa9 in Pre-S1 in sample 498 and aa201 in the S gene in sample 716 (data not shown).

## Discussion

The majority (94.34%, 200/212) of the HBsAg−/HBV NAT+ blood samples was HBV-infected, 81.13% (172/212) of which were identified as OBIs (Table 1). Samples not confirmed as HBV DNA positive may be related to (a) viral load under the confirmatory assay detection limit, (b) NAT screening false positive, or (c) genetic variability. Donors with OBI had normal ALT levels, detectable anti-HBc and low viral loads (median: 71.15 IU/ml, 62.21% cases were lower than 100 IU/ml) which was consistent to previous studies^8,22^. Incomplete control of HBV replication by the host immunity may lead to low viremia. Anti-HBs formed by the selection of HBsAg escape mutations spontaneously and the consequences for vaccination of the general population against HBV^28,30^ may facilitate the incomplete immune control. OBI is related to the antiviral immune response, which is believed to be important for maintaining HBV control^8^. The immune system effectively controls HBV (even if it is not cleared) in most OBI cases^31^. Nearly half of these individuals (40.70%, 70/172) carried detectable anti-HBs in this study, which was similar to what was reported in OBIs in South Africa and Europe (45%)^17,18^. It is known that anti-HBs are considered a protective antibody and proof that people have developed immunity. So, OBI with anti-HBs may indicate incomplete immunity.

In previous studies, OBI-related mutations are often identified without matched control^21,32^. Consequently, it was impossible to rule out natural polymorphisms and/or differences related to the tissue source of the virus, the clinical status of the HBV infected persons, or the geographic origin of individuals^32^. We selected matching sequences obtained from HBsAg+ asymptomatic and apparently healthy blood donors identified during the same blood screening process in Guangzhou Blood Center as control groups in order to overcome limitations above.

Naturally occurring mutations in the HBV genome have been attributed to play an important role in the persistence of HBV infection. Significantly higher mean nucleotide variability was observed in the SP2 and CURS regulatory regions only in the OBI_B_ strains sequences than controls in this study. However, the regulatory regions were conserved for OBI_C_ strains compared to HBsAg+ strains, but a significantly higher nucleotide variability was observed in the SP1, SP2, ENH1, and ENH2 regulatory regions in OBI_C_ strains compared to the HBsAg+ strains in previous studies^22,33^. The reason for the discrepancy may be related to (a) different study populations, (b) our more reliable control from the same population infected with HBV.

The mean number of amino acid substitutions in Pre-S/S regions from OBI_B_ strains sequences was higher than in their wild-type strains, and this phenomenon was the same in OBI_C_ strains sequences (except for Pre-S1; Table 2). The genome's Pre-S/S ORF encodes the three envelope glycoproteins, which are produced by differential translation initiation at each of three in-frame start codons. The three envelope glycoproteins are called the large (L), middle (M), and small (S) HBsAgs. The expression of envelope protein is essential for virion assembly and secretion. Therefore, deletions, insertions, and point mutations may interfere with HBsAg detection in Pre-S/S.

Mutations in the Pre-S1 and Pre-S2 gene may reduce the expression of L-HBsAg and M protein, respectively. In this study, deletions and some mutations happened in Pre-S1 and Pre-S2 region. Moreover, one ps1C25T mutation resulted in a stop codon in the Pre-S1 gene in sample 498. The specific ratio of L-HBsAg and S-HBsAg protein is essential for assembly of the envelop particles because an excessively low or high ratio of L/S proteins could change assembly and secretion of HBsAg, and reduce secretion of virion^34^. HBsAg secretion is significantly reduced, envelope proteins are retained in the endoplasmic reticulum, virion secretion efficiency is reduced, and nuclear accumulation of higher amounts of covalently closed circular DNA in Pre-S variant HBV compared with in wild-type HBV^35^.

The factors of occult hepatitis B infection are complicated and not yet been fully elucidated. Mutations in S gene may contribute to occult infection. The S protein corresponds to HBsAg^36^. Mutations in the S gene may affect immunogenicity, antigenicity, expression, and/or secretion of HBsAg, causing HBsAg test failed^37,38^, reducing the replication and/or secretion of the virion, exerting a negative effect on HBsAg^14,39^, or avoiding final clearance by the immune system and finally leading to OBI. HBV DNA level strains with mutations sG145R (pR499Q) was significantly lower than strains without the mutation, so was mutation sI226S in OBI_B_ strains. However, further functional studies are needed. In this study, an insertion in sample 170 and the new OBI-point mutation sT118K in gtC strains were all located in MHR. MHR is the most important antigenic determinant of all HBV strains and is crucial to the HBsAg detection and HBV vaccines development^14,36^. An aa201 stop codon mutation in OBI_C_ sample 716 was found in the S gene. Some new OBI-point mutation in the S gene are uncommon mutations in this study. Among them, mutation sT118K in MHR caused a decrease in the thermo-stabilities (0.31 kcal/mol), which might lead to structural instability under the thermal circumstance. The prediction of the secondary structures showed that the proportion of beta-turns, and random coils changed after sT118K mutation. This change may influence the 3D structures of S protein and then affect biological function, supported by protein 3D structures. Especially, beta-turns are generally located on the surface of proteins and are related to molecular recognition, so reduced beta-turns upon mutation sT118K may influence the detection function of the HBV diagnostic ELISA kit. Strains with mutations sG145R (pR499Q) and sI226S had lower HBV DNA level, which may imply that the mutation in S gene may reduce the replication and/or secretion of the virion. However, further functional studies are needed.

Mutations in the Pol (RT) gene may be one of the reasons for the low titers of most OBI cases. The RT activity is important for the replication of HBV DNA^40^. The mean number of amino acid substitutions in the Pol region (*P* = 0.009 and *P* = 0.042), especially in the RT region (*P* < 0.001 and *P* = 0.005) from OBI_B_ and OBI_C_ sequences, was higher than in their wild-type strains (Table 2), which was similar to a previous study^41^. Seven new OBI-related mutations were identified gtB, and three in gtC strains in polymerase gene in the present study. Most of these mutations were located in the RT region. Mutation pR499Q (rtR153Q) and pH580Q (rtH234Q) corresponding to sG145R and sI226N/S in OBI_B_ strains, respectively, and pH468N (rtR122 Q) and pH472Q (rtH126Q) corresponding to sS114T and sT118K in OBI_c_ strains, respectively, were observed in this study. Some studies have a focus on the concomitant mutations in RT and S region about drug-resistant and vaccine-escape due to their overlapping protein-coding regions^42,43^.

It is reported that the mutations of the core protein in HBV infection is not only related to the low secretion of HBV virions, but also related to immune escape epitopes at CTL and Th levels and severe liver disease (such as liver cirrhosis and hepatocellular carcinoma)^44–46^. One new OBI-related mutation, T147A in the core gene, was identified in OBI_B_ strains. The mutations of the OBI core protein in CTL epitope cluster 141–151 may become an escape epitope. Although these mutations may reduce the adaptability of the virus, it still contributes to the persistence of HBV infection^46^. Studies have recently shown that the HBc (9-residues, 141–149) linker peptide between the N-terminal domain and C-terminal domain, which plays a key role in multiple stages of virus replication rather than just as a spacer with no specific function and strongly implicated the HBc linker in recruiting the protein phosphatase 2A and other host factors to regulate multiple stages of HBV replication^47,48^, which may result in low viremia.

OBI is the potential risk of HBV transmission through blood transfusion (the minimal infectious dose is 3 IU/ml), organ transplantation, and from occult infected mothers to newborns^9–11^, and episodes of reactivation can occur after the development of an immunodeficiency, and then acute hepatitis and occasionally fulminant hepatitis may happen after reactivation^49^. The reactivation can make the progression of liver damage, resulting in fibrotic conditions that promote the development of cirrhosis^50^. The study of characteristics of a large sample size of the full-length genome of OBI may better to understand the situation of OBI infections in blood donors and further help us to pay attention to the fact that reactivation of OBI strains occurs.

In conclusion, OBI maintained by host, viral, immunological, and/or epigenetic factors, is one of the most challenging clinical features in the viral hepatitis study^50^. We conducted a comprehensive survey about the characteristics of a large sample size of the full-length genome of OBI. The variabilities and mutations mainly occurred in the Pol and Pre-S/S region both in OBI_B_ and OBI_C_ strains, which may lead to HBsAg undetectable and low HBV DNA viral load in the present study, but the relationship remains to be confirmed by functional studies, which are being planned.

## Materials and methods

### Sample identification

HBsAg and antibodies to human immunodeficiency virus (HIV), hepatitis C virus (HCV), and Treponema pallidum (TP) were tested by individual donation enzyme immunoassays (EIAs) testing. The qualified blood donors were further screened for HBV, HCV, and HIV genomes by NAT with Procleix Ultrio Plus multiplex Assay, and then with HBV Discriminatory Assay (Grifols Diagnostic Solutions, Inc.) on the Tigris platform; the lower detection limits of the two NAT assays were 3.4 IU/ml and 4.1 IU/ml, respectively. Two hundred and twelve HBsAg−/HBV NAT+ blood donors were enrolled in Guangzhou Blood Center from March 2015 to May 2017. The diagnostic criteria of OBI are described in detail in previous studies^17,22^. Briefly, samples identified as OBI were confirmed by combining real-time quantitative polymerase chain reaction (Q-PCR), nested amplification, anti-HBc, and anti-HBs, excluding the samples of the window period, false positive, and convalescent period of acute infection. All participants were duly informed about this study, and written informed consent was obtained from each participant. All procedures performed in this study involving human participants were in accordance with the 1964 Helsinki declaration and its later amendments or comparable ethical standards. This study was approved by the Medical Ethics Committee of Guangzhou Blood Center.

### HBV serological testing

HBV serologic markers (HBsAg, anti-HBs, HBeAg, anti-HBe, and anti-HBc) were analyzed by a highly sensitive electrochemiluminescence immunoassay [(ECLIA), cobas e602; Roche Diagnostics, Mannheim, Germany] according to the manufacturer's instructions. The limit of ECLIA for HBsAg is 0.05 IU/ml.

### HBV DNA quantification, amplification, sequencing, and phylogenetic analysis

The HBV DNA from the HBsAg-/HBV NAT+ samples was extracted from 2.5 mL of plasma, using a large volume of high-purity virus nucleic acid extraction kit (Roche Diagnostic, Germany)^51^. Q-PCR was used to quantify viral load (sensitivity 5 IU/ml). Viral DNA was also used to amplified by nested PCRs for an HBV long fragment [the HBV full length-genome minus 53 bp, nucleotide (nt)1804 to 1856] and the HBV basic core promoter/precore gene (BCP/PC, nt 1679 to 1973), as described previously^18,22,51^. Samples positive with Q-PCR or nested PCR tests were considered HBV DNA positive. Samples negative with Q-PCR and nested PCR tests were considered HBV DNA not confirmed. Blood donors with anti-HBc or HBV DNA testing negative were used in follow-up analyses.

The HBV long PCR fragments products were ligated into a cloning vector, which was used for the transformation of *E. coli*, followed by cultivation overnight and five clones were picked up for sequencing. We obtained the full-length genome sequence by combining the two overlapping fragments. MAFFT version 7 (https://mafft.cbrc.jp/alignment/software/) was used to generate the multiple sequences alignment. The phylogenetic tree was constructed using MEGA X software (www.megasoftware.net) on the maximum-likelihood method. The reliability of the tree was estimated using 1000 bootstrap replications. HBV subgenotype reference sequences^52^ were downloaded from the National Center for Biotechnology Information (NCBI) database (https://www.ncbi.nlm.nih.gov/nuccore/). HBV genotypes/subgenotypes were confirmed by the phylogenetic tree.

### Occult HBV sequence analyses

A consensus sequences was from each OBI plasma. Consensus sequences were used for alignment analyses^18^. BioEdit software (https://bioedit.software.informer.com/) was used to calculate the intragroup variability based on “Sequence difference count Matrix”. HBV wild-type sequences were used as controls, which were obtained from HBsAg-positive (HBsAg+) strains selected from blood donors in Guangzhou Blood Center. The whole genomes of 81 control strains (58 gtB and 23 gtC) were amplified successfully, as described previously^53^. A phylogenetic tree of control sequences was constructed similarly to the sequences of OBI strains (Supplementary Fig. S1). Average intragroup variability was calculated as the number of nucleotides (regulatory region sequences) or amino acid (protein sequences) substitution differences between OBI strain sequences and control HBV strain sequences of different genotypes. Point mutations were investigated between OBI and control group throughout the four open reading frames (ORFs, Pre-S/S, Pol, Pre-Core/Core, and X region). The OBI-related point mutations in OBI sample sequences that were not present in any of the reference isolates were designated as uncommon mutations^33^. The secondary structures of the S protein were predicted by SOPMA (https://npsa-prabi.ibcp.fr/cgi-bin/npsa_automat.pl?page=npsa_sopma.html). GenBank accession numbers of the full-length HBV genomic sequences from 81 HBsAg+ and 50 OBI blood donors in this study were OM669567 through OM669697.

### Statistical analysis

Intergroup variability analyses were performed by the non-parametric Mann–Whitney test. HBV DNA level between strains with OBI-related mutations and strains without OBI-related mutations were compared by T-test, and the non-parametric Mann–Whitney test was used when the condition of T-test was not satisfied. The significance of differences in point mutations between OBI and control group were determined using the Fisher's exact test. All tests used were two-tailed. All statistical analysis was performed by SPSS 22.0 software (SPSS, Chicago, IL, USA). A *P*-value of < 0.05 was considered to be statistically significant.

## Supplementary Information

Supplementary Information.

## Data Availability

The datasets generated during the current study are available in the National Center for Biotechnology Information Search database repository (NCBI) (https://www.ncbi.nlm.nih.gov/nuccore/). GenBank accession numbers were OM669567 through OM669697.

## Abbreviations

HBV: Hepatitis B virus
OBI: Occult hepatitis B virus infection
SP2: Pre-S2/S promoter region
CURS: Core upstream regulatory sequence
aa: Amino acid
ORF: Open reading frames
NAT: Nucleic acid test
HIV: Human immunodeficiency virus
HCV: Hepatitis C virus
TP: Treponema pallidum
EIA: Enzyme immunoassay
Q-PCR: Real-time quantitative polymerase chain reaction
ECLIA: Electrochemiluminescence immunoassay
BCP/PC: Basic core promoter/precore gene
