# Supplementary material for: Molecular characteristics of the full-length genome of occult hepatitis B virus from blood donors in China
Source: Sci Rep. 2022 May 17;12:8194. doi: 10.1038/s41598-022-12288-0 (PMC9114411; doi:10.1038/s41598-022-12288-0)
Supplement: Supplementary file 1 — Supplementary Information. [file 41598_2022_12288_MOESM1_ESM.pdf]

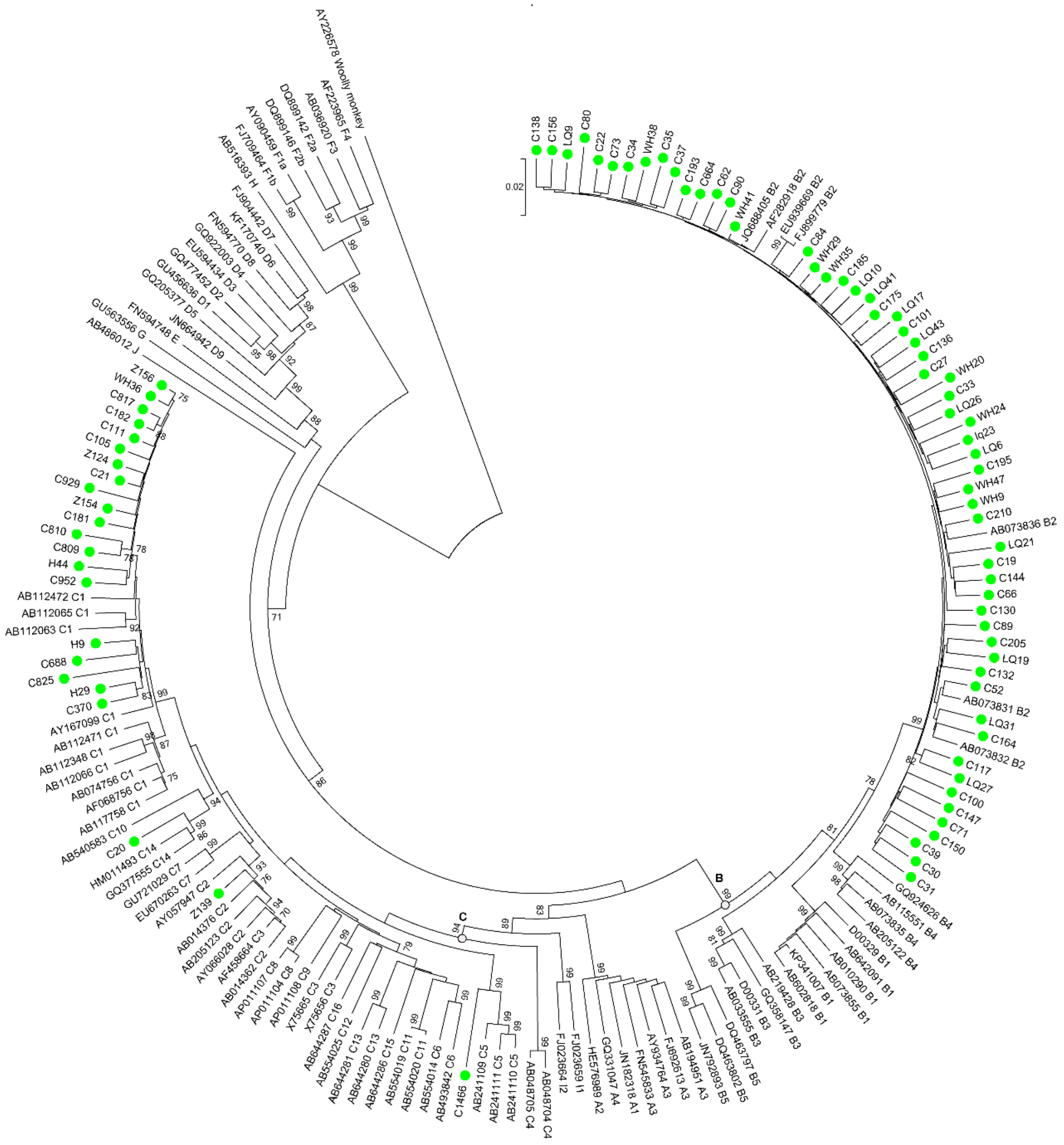

Figure S1. Estimated maximum-likelihood phylogeny for full-length genome sequences of 58 HBsAg+ control strains controls. Green circles indicate controls in this study, and the rest indicate reference sequences of proposed genotypes and subgenotypes. All genotype B control strains were classified to subgenotype B2. Twenty genotype C control strains were subgenotype C1 and 3 belonged to other C subgenotypes. Bootstrap analysis values (>70%) are displayed on the branches. The bars at the middle top of the figure show the scale in nucleotide substitutions per site. AY226578: from woolly monkey as an out-group.

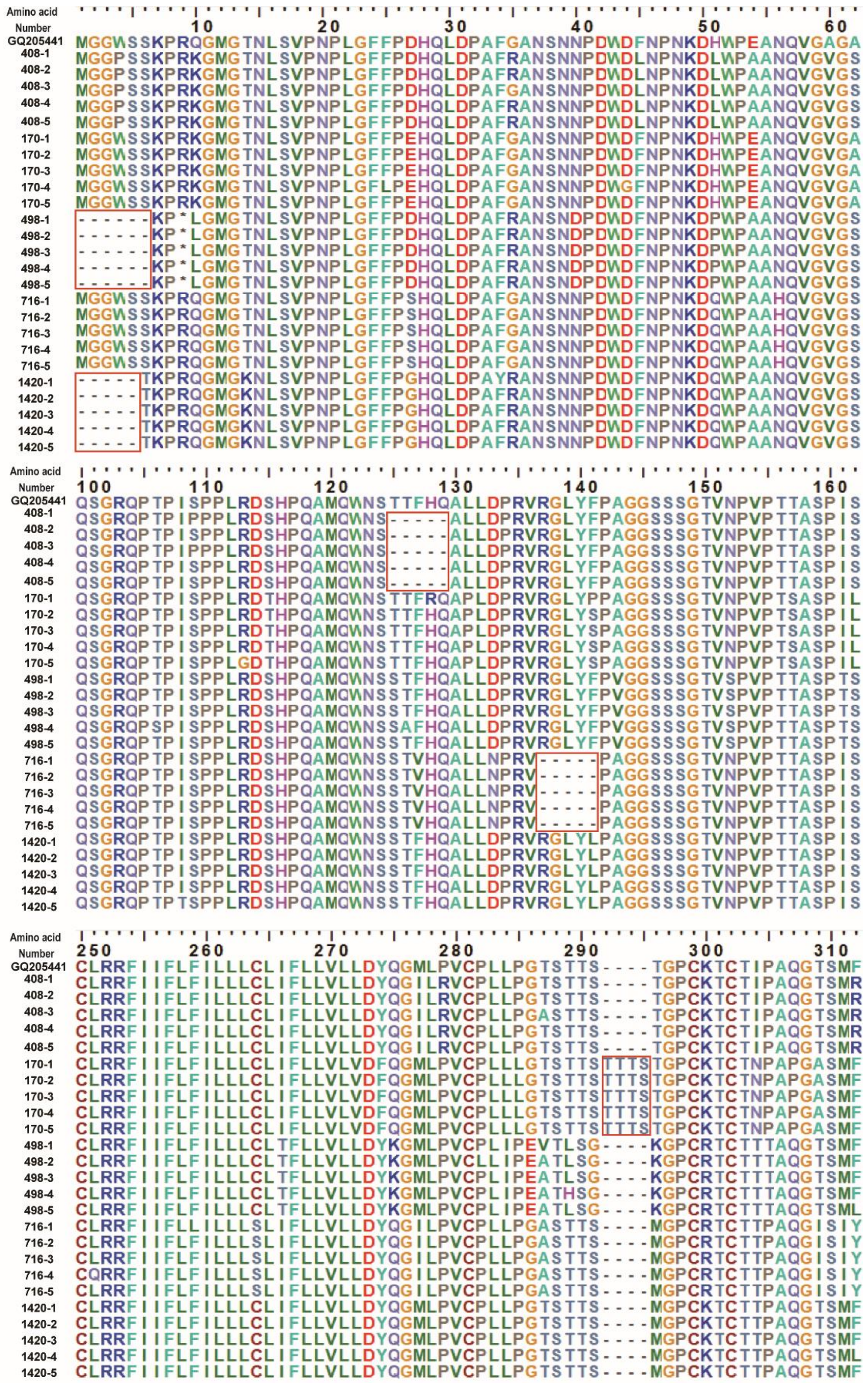

Figure S2. Amino acid sequences of PreS/S region for samples with deletion and insertion. Deleted or inserted amino acids were indicated within red boxes. Five clones for each sample were analyzed.

Table S1. HBV DNA viral loads of blood donors confirmed as HBV DNA positive

		Unquantifiable	<100 (IU/ml)	100-200 (IU/ml)	≥200 (IU/ml)	Total
OBI		28	79	28	37	172
	Genotype B	2	12	6	13	33[Φ]
	Genotype C	5	3	2	7	17[Φ]
Other[§]		0	2	1	3	6*
	Genotype B	0	1	1	1	3[Φ]
	Genotype C	0	0	0	1	1[Φ]
Not classifiable		2	4	2	14	22
	Genotype B	1	0	1	7	9[Φ]
	Genotype C	0	1	0	2	3[Φ]

[*]These 6 samples were positive for HBV DNA (qPCR or nested PCR positive) and negative for anti-HBc, however, these follow-up samples were negative for HBV DNA by NAT, qPCR and nested PCR assays. Index sample 73, 368, 987 contained 29.3 IU/L, 84.3 IU/L, <2 IU/L of anti-HBs but the first follow-up sample contained 239.1 IU/L, 75.7 IU/L, 69.5 IU/L of anti-HBs, respectively. The rest three samples were anti-HBs negative but the follow-up samples contained >1000 IU/L of anti-HBs. These index samples with non-reactive for anti-HBc were classified as recent HBV infection, and acute resolving may happen to these 6 blood donors.

[Φ] In OBI, other, and not classifiable groups, we got 50, 4 and 12 full-length genome sequences, respectively. In total, only 66 strains were genotyped successfully.

Table S2. OBI-related mutations except novel mutations of HBV genome relating to the genotype B and C

Region	Mutation	Frequency in OBI blood donors (%)	Frequency in HBs-positive blood donors (%)	*p*-Value（OBI vs HBsAg-positive）
Gt-B				
Pres2	R16K	3/33 (9.09)	0/58 (0)	0.042
	L46S	5/33 (15.15)	2/58 (3.33)	0.047
S	E2G	5/33 (15.15)	0/58 (0)	0.003
	E2A	4/33 (12.12)	0/58 (0)	0.009
	Q101R	4/33 (12.12)	0/58 (0)	0.013
	P105R	4/33 (12.12)	0/58 (0)	0.014
	M133T	5/33 (15.15)	1/58 (1.72)	0.013
	M133I	4/33 (12.12)	0/58 (0)	0.009
	G145R	3/33 (9.09)	0/58 (0)	0.036
	S167L	5/33 (15.15)	0/58(0)	0.005
	V168A	11/33 (33.33)	2/58 (3.45)	<0.001
	S174N	8/33 (24.24)	2/58 (3.45)	0.004
	L175S	6/33 (18.18)	0/58 (0)	0.002
	V177A	8/33 (24.24)	0/58 (0)	0.0001
	F220C	8/33 (24.24)	4/58 (6.67)	0.020
	I226S	3/33 (9.09)	0/58 (0)	0.030
Core	P50H	8/33 (24.24)	3/58 (6.90)	0.023
Pol	R499Q$^{\delta}$	3/33 (9.09)	0/58 (0)	0.040
	H580Q$^{\delta}$	8/33 (24.24)	0/58 (0)	<0.001
Gt-C				
S	Q101R	3/15 (40.00)	0/23 (0)	0.037
	Q101K	3/15 (40.00)	0/23 (0)	0.037
	S114T	4/15 (26.67)	0/23 (0)	0.018
	K122R	3/15 (20.00)	0/23 (0)	0.047
	S174N	4/15 (26.67)	0/23 (0)	0.018
	L175S	4/15 (26.67)	0/23 (0)	0.021
Pol	H468N$^{\delta}$	3/12 (25.00)	0/23 (0)	0.037

$^{\delta}$ Mutations pR499Q and pH580Q is corresponding to sG145R and sI226N/S in OBI_B strains, and pH468N is corresponding to sS114T in OBIc strains.

Table S3. Comparison of viral load between in OBI-related mutation cases and in non-OBI-related mutation cases

Region	Mutation	OBI-related mutation cases Mean±SD	Non-OBI-related mutation cases Mean±SD	*p*-Value
Gt-B				
Pres1	E39K	2.36±0.56	2.02±0.70	0.272
	E39D	2.22±0.56	2.09±0.70	0.757
	S101T	2.04±0.68	2.10±0.69	0.877
Pres2	Q10R	2.57±0.31	2.09±0.71	0.261
	R16K	1.97±0.22	2.11±0.71	0.739
	L46S	2.13±0.24	2.09±0.73	0.914
S	E2G	1.68±0.91	2.17±0.63	0.140
	E2A	2.19±0.34	2.19±0.372	0.784
	Q101R	1.56±0.95	2.17±0.63	0.093
	P105R	1.75±0.97	2.15±0.64	0.274
	M133T	2.14±0.55	2.09±0.71	0.875
	M133I	2.38±0.55	2.06±0.69	0.376
	**G145R**	1.11±0.63	2.20±0.61	**0.006**
	S167L	2.01±0.31	2.11±0.73	0.575
	V168A	2.26±0.44	2.02±0.77	0.355
	S174N	2.02±0.53	2.07±0.73	0.648
	L175S	2.20±0.65	2.08±0.70	0.697
	V177A	2.26±0.47	2.05±0.74	0.461
	P178Q	1.67±1.17	2.14±0.71	0.261
	Q181R	1.98±0.56	2.12±0.70	0.540
	F220C	2.02±0.85	2.12±0.63	0.710
	I226N	2.04±0.52	2.11±0.71	0.858
	**I226S**	1.29±0.78	2.18±0.63	**0.027**
Core	P50H	2.11±0.83	2.10±0.64	0.977
	T147A	2.00±0.77	2.14±0.65	0.585
Pol	G261R	1.89±0.63	2.13±0.69	0.377
	V281D	2.04±0.68	2.10±0.69	0.877
	R364K	1.66±0.36	2.16±0.70	0.093
	V458I	2.12±0.83	2.10±0.67	0.979
	N470H	2.67±0.29	2.04±0.68	0.126
	**R499Q**$^{\delta}$	1.11±0.63	2.20±0.61	**0.006**
	N572T	2.08±0.13	2.10±0.71	0.898
	H580Q$^{\delta}$	1.73±0.66	2.27±0.66	0.082
	Q613H	2.10±0.46	2.10±0.73	0.874
Gt-C				
S	Q101R	1.227±1.44	2.20±1.07	0.365
	Q101K	2.43±0.22	1.90±1.29	0.633
	S114T	2.22±0.64	1.93±1.32	0.851
	T118K	2.02±1.23	2.00±1.20	0.981
	K122R	1.67±1.16	2.09±1.20	0.587
	S174N	2.23±0.77	1.89±1.29	0.661
	L175S	2.03±1.25	2.20±1.20	0.973

Pol	R119L	2.00±1.17	1.22±1.44	0.482
	H468N [δ]	2.08±0.70	1.72±1.38	0.864
	H472Q[δ]	2.06±1.26	1.63±1.26	0.567
	I615L	2.56±1.87	1.55±0.94	0.282

[δ]Mutations pR499Q and pH580Q are corresponding to sG145R and sI226N/S in OBIB strains, and pH468N and pH472Q are corresponding to sS114T and sT118K in OBIc strains.
